# Addition of Dexmedetomidine to Propofol Anesthesia for Middle-Ear Surgeries: A Prospective Randomized Double-Blind Study

**DOI:** 10.7759/cureus.68025

**Published:** 2024-08-28

**Authors:** Swathi Kumari K., H.G. Thippeswamy, Shruthi R Nayak, Shrirang V Torgal

**Affiliations:** 1 Anaesthesiology, Yenepoya Medical College and Hospital, Mangalore, IND; 2 Anaesthesiology, SDM College of Medical Sciences and Hospital, Dharwad, IND

**Keywords:** anesthesia adjuvants, intravenous anesthesia, balanced anaesthesia, controlled hypotension, propofol, dexmedetomidine

## Abstract

Background

Middle-ear surgery commonly performed under a microscope requires a bloodless field provided by hypotensive anesthesia. Our objective was to study the effects of dexmedetomidine on propofol consumption and intraoperative hemodynamic stability.

Methods

One hundred adults undergoing elective middle-ear surgery were randomized into two groups. The propofol+dexmedetomidine group (Group PD) received a loading dose of dexmedetomidine 1μg/kg in 10ml normal saline over 10min followed by infusion of the same at 0.5μg/kg/h. Propofol-only group (Group P) received 10ml normal saline over 10min followed by an infusion of the same. General anesthesia was induced with intravenous morphine, propofol, and vecuronium, and maintained with propofol, oxygen, and N_2_O. During microscope use, we aimed to maintain mean arterial pressure (MAP) within 60-69mmHg.

Results

There was no significant difference in the mean (SD) consumption of propofol [Group P 8.6 (2.1)mg/kg/h vs Group PD 8.1 (1.5)mg/kg/h, *P*=0.172]. The induction dose of propofol was significantly less in Group PD [1.8 (0.3) vs 2 (0.4)mg/kg, *P*<0.001]. Except for the baseline value, the heart rate was significantly lower in Group PD, *P*<0.001. The time duration during which MAP was within 60-69mmHg was higher in Group P [37.5 (36.8) vs 30.9 (38.3)min] though the difference was not statistically significant. The recovery was delayed in Group PD [25.4 (8.6) vs 17.6 (4.9)min, *P*<0.001]. Group PD had a significantly better operative field, *P*=0.0003.

Conclusion

The addition of dexmedetomidine did not reduce propofol consumption but reduced the induction dose of propofol. Propofol and dexmedetomidine combination provided comparable mean arterial pressure and better operative field but caused delayed recovery.

## Introduction

Dexmedetomidine is an alpha-2 adrenoreceptor agonist approved by the U.S. Food and Drug Administration in 1999 for use in the intensive care unit (ICU) due to its sedative and analgesic properties [1]. Owing to its central sympatholytic effect, dexmedetomidine blunts hemodynamic responses in the perioperative period [2].

Middle-ear surgeries are commonly performed under general anesthesia (GA) using an operating microscope and require a bloodless operative field [3]. Hypotensive anesthesia has facilitated the practice of middle-ear surgery by providing an optimal operative field achieved by adequate analgesia, a deeper plane of anesthesia, and a stable heart rate (HR) and blood pressure (BP) [4]. Conventionally, anesthesiologists use higher doses of potent opioids, anesthetic agents, and sometimes vasodilators and negative inotropic agents. However, this approach has some disadvantages, such as delayed recovery, respiratory depression, and a higher incidence of postoperative nausea and vomiting (PONV). Dexmedetomidine is said to potentiate the action of opioids and analgesics, thereby reducing the need for large doses of these drugs [5].

In this work, we aimed to evaluate the effect of the addition of dexmedetomidine to propofol in patients undergoing middle-ear surgery. We hypothesized that the addition of dexmedetomidine to propofol anesthesia would reduce the requirement of propofol, and provide stable hemodynamics and a better surgical field, with faster recovery from GA. The primary outcome measured was intraoperative consumption of propofol. The secondary outcomes measured were induction dose of propofol, time duration of mean arterial pressure (MAP) within the target range of 60-69 mmHg during microscope use, the quality of the operative field, recovery time, sedation and pain scores in the post-anesthesia care unit (PACU), and adverse events such as PONV and respiratory depression.

## Materials and methods

We conducted a prospective randomized double-blind observational study in the Department of Anaesthesiology, SDM College of Medical Sciences and Hospital, Sattur, Dharwad, Karnataka, India, from November 2012 to August 2014. We enrolled 100 patients in the age group of 18-60 years, belonging to the American Society of Anesthesiologists Physical Status (ASA PS) Class-I or II, who were undergoing elective middle-ear surgery under GA lasting more than two hours. Patients on β-blockers or vasodilator therapy, those having arrhythmias or valvular heart disease, those allergic to the medications being used, pregnant women, and those who refused consent were excluded. The study was conducted after receiving permission from the relevant Institutional Ethics Committee (SDMIEC:395:2012) and informed consent from all enrolled patients.

All patients were kept nil-per-oral overnight and received oral ranitidine 150 mg the night before and on the morning of surgery. Sedative premedication was not administered to any patients. The simple randomization method was followed. The patients were randomly allocated to either the propofol-only group (Group P) or the propofol + dexmedetomidine group (Group PD) using computer-generated random numbers. Sealed envelopes were used, which were opened just before shifting the patient to the operating room (OR). Anesthetic drugs were prepared by the anesthesiologist who opened the envelope but was not involved in the intraoperative management or postoperative assessment of the patients. Once the envelope was opened in the OR, drugs were loaded and kept ready, the patient was shifted into the OR.

In the OR, after securing appropriate intravenous access monitoring via electrocardiogram, non-invasive oscillometric blood pressure and pulse-oximeter devices were initiated, and baseline values were recorded. Patients allocated to Group PD received an initial loading dose of dexmedetomidine 1 μg/kg prepared in a 20-mL syringe and 10 mL of normal saline given over a period of 10 min followed by an infusion of dexmedetomidine (4 μg/mL) at 0.5 μg/kg/h until the end of microscope use. Patients allocated to Group P received 10 mL of normal saline from a 20-mL syringe given over 10 min followed by an infusion of saline at the calculated rate until the end of microscope use. Then the patients received intravenous morphine 0.1 mg/kg and were pre-oxygenated for 3 min. GA was induced with propofol infusion beginning with a rate of 40 mg/kg/h until the loss of verbal contact, then 20 mg/kg/h for the next 1 min, and 10 mg/kg/h until intubation. Intravenous vecuronium 0.1 mg/kg was administered, and the trachea was intubated with an endotracheal tube. Ventilation was controlled, and end-tidal carbon dioxide was maintained between 35 and 40 mmHg.

Anesthesia was maintained with propofol and 50% oxygen in N2O. Following intubation, we attempted to achieve the target MAP of 60-69 mmHg by adjusting the propofol infusion (starting dose = 100 μg/kg/min) until the use of the microscope. The size of the bolus was 0.25 mg/kg every 5 min followed by an increase in infusion rate by 25 μg/kg/min until the target MAP was achieved or the maximum infusion rate was 150 μg/kg/min, whichever occurred earlier. In the case of MAP<60 mmHg, the propofol infusion was stopped for 1 min and restarted at a rate decreased by 25 μg/kg/min. During surgery under the microscope, the time duration during which MAP remained within the target range of 60-69 mmHg was noted. After the microscope was withdrawn, the infusion of propofol was adjusted to gradually bring the MAP to >70 mmHg. The patients received vecuronium top-ups (1/5th of the intubating dose) every 30 min, and additional morphine 0.05 mg/kg if the surgery lasted more than 4 h. Intravenous ephedrine 6 mg was administered if MAP was <60 mmHg. An HR of <40 beats/min was treated with intravenous atropine 0.3 mg.

Nitrous oxide and propofol infusion were stopped at the application of the final skin suture, and neuromuscular blockade was reversed with intravenous neostigmine (50 μg/kg) and glycopyrrolate (10 μg/kg). The trachea was extubated upon the return of consciousness. The time to recovery, defined as the time from stopping of propofol infusion until spontaneous eye opening/ response to verbal commands, was noted. The operating surgeon was asked to rate the operative field as “excellent,” “satisfactory,” or “unsatisfactory.”

All patients were assessed for level of sedation, analgesia, PONV, and respiratory depression on arrival to the PACU, and again at 30 min, 1 h, and 2 h from the time of arrival to the PACU. Sedation was assessed using the Ramsay Sedation Assessment Scale (RSS). Postoperative analgesia was assessed using an 11-point numerical rating score (NRS), where 0=no pain and 10=worst imaginable pain. Intravenous morphine 0.05 mg/kg was administered if the patient complained of pain and had NRS>3. Intravenous ramosetron 0.3 mg served as a rescue antiemetic in the PACU. Consumption of morphine and ramosetron in the PACU was noted. The patients, anesthesiologist, surgeon, and the person who did postoperative follow-up were blinded to the group allocation.

Chi-squared tests were used to compare the two groups in terms of ASA PS, surgical field, NRS, and RSS scores. Independent t-tests were used to compare the means between the groups. Paired t-tests were used to compare the MAP and HR response to intubation among the groups. Results for continuous measurements are presented as means (standard deviations; SDs), and categorical measurements are presented as numbers (percentages). P<0.05 was considered statistically significant. All statistical analyses were performed using Statistical Package for Social Sciences (SPSS) version 21.0 (IBM Corp., Armonk, USA). The sample size was estimated based on a pilot study, which showed a mean propofol consumption of 8 (2.1) mg/kg/h in Group P and 7.5 mg/kg/h in Group PD. Based on a study done by Bakhamees HS et al., considering a power (β) of 90%, α = 5%, and a 20% difference in propofol consumption considered significant, each group required a sample size of 46 [1].

## Results

**Figure 1:**
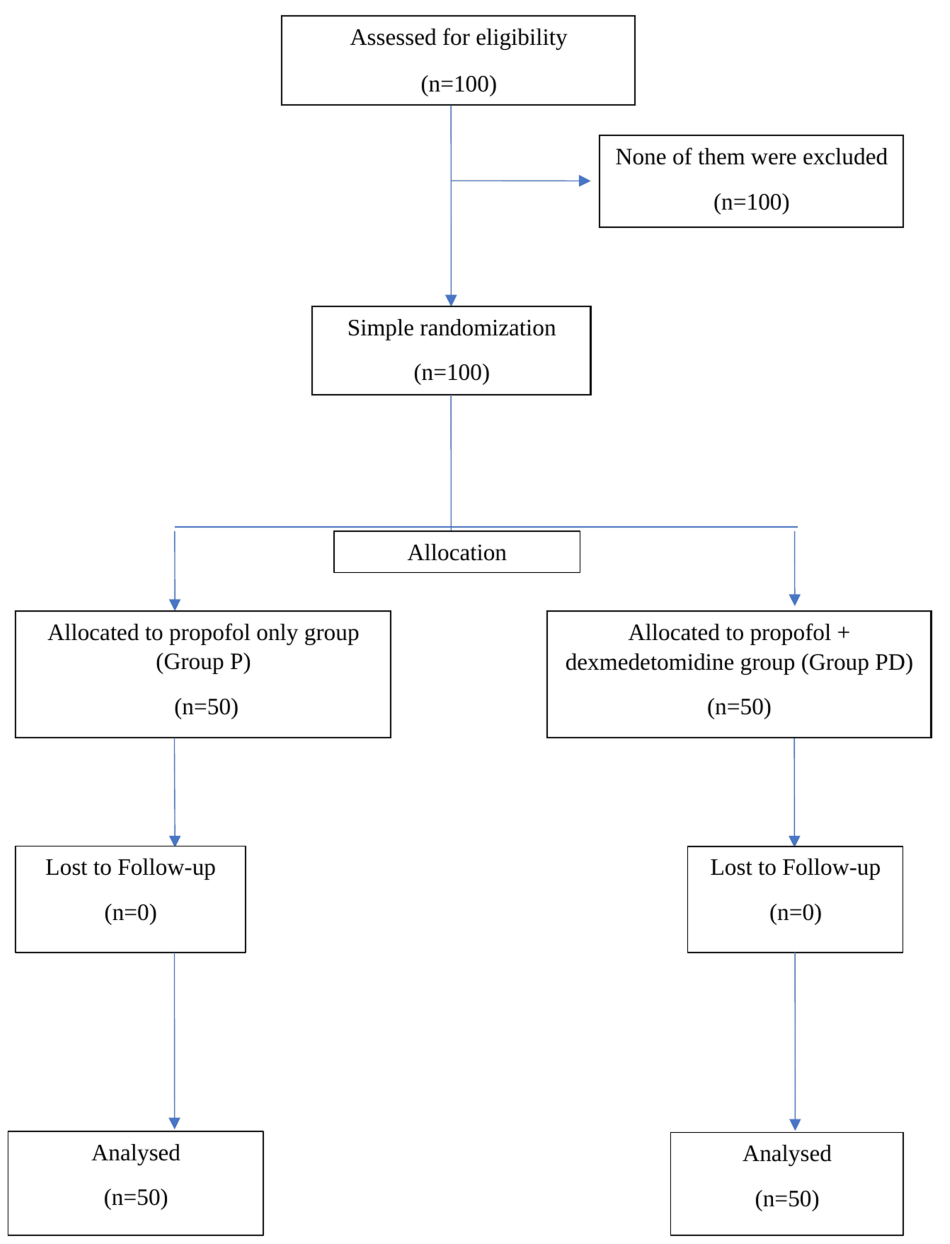
CONSORT chart of simple randomization CONSORT: Consolidated Standards of Reporting Trials

In this prospective randomized study, one hundred patients were assessed for eligibility and randomly assigned to two groups: Group P (propofol only group) and Group PD (propofol+dexmedetomidine group), each consisting of 50 participants. All patients successfully completed the study (Figure 1).

Demographic data (Table 1), duration of anesthesia and surgery, and total microscope time were comparable between the two groups (Table 2).

**Table 1:** Patient characteristics Values presented as means (standard deviations) except *gender and ASA PS (American Society of Anesthesiologists Physical Status), which are presented as numbers (percentage)

Parameter	Group P (n=50)	Group PD (n=50)	p-value
Age (years)	29.6 (10.7)	30.3 (9.9)	0.859
Weight (kg)	56 (11.4)	57.6 (11.6)	0.794
Body Mass Index (kg/m^2^)	22 (3.9)	22.8 (3.7)	0.179
Gender* (Male/Female)	25 (50)/25 (50)	23 (46)/27 (54)	0.688
ASA PS* (I/II)	40 (80)/10 (20)	38 (76)/12 (24)	0.629

**Table 2:** Propofol and morphine consumption, duration of surgery and microscope use, duration of anesthesia and recovery time Values presented as means (standard deviations)

Parameter	Group P (n=50)	Group PD (n=50)	p-value
Induction dose of propofol (mg/kg)	2 (0.4)	1.2 (0.3)	<0.001
Total propofol consumption (mg/kg/h)	8.6 (2.1)	8.1 (1.5)	0.172
Morphine consumption (mg)	6.2 (1.3)	6.3 (1.5)	0.753
Anesthesia duration (min)	193.1 (53.2)	193 (49.1)	0.992
Surgery duration (min)	167.4 (50.8)	168.1 (48.1)	0.944
Duration of microscope use (min)	139.5 (49.4)	143.1 (43.6)	0.700
Recovery time (min)	17.6 (4.9)	25.4 (8.6)	< 0.001

Patients in Group PD required a significantly lower induction dose of propofol when compared to those in Group P (p<0.001). However, total consumption of propofol and morphine was comparable between the two groups. The recovery time was significantly prolonged in Group PD when compared with Group P (p<0.001).

HR, except for the baseline value, was significantly lower in Group PD when compared with Group P during the intraoperative period (p<0.001). For within-group comparisons, HR just before intubation, when compared to baseline values, was significantly higher in Group P, whereas it was significantly lower in Group PD. Group PD had significantly lower HR for up to 4 min post-intubation when compared to HR just before intubation (Table 3).

**Table 3:** Comparison of heart rate (beats/min) between groups LA: Local anesthesia. Values presented as means (standard deviations). p-value comparisons are between the two groups (unpaired t-test). p-values* for within-group comparison (paired t-test), **when compared to baseline, and #when compared to just before intubation.

	Group P	P value*	Group PD	P value*	P value
Baseline	83.6 (16.2)		81.3 (14.2)		0.477
After loading dose	83.6 (16)		65.9 (14.6)		<0.001
Induction	83.7 (16.1)		67.8 (14.6)		<0.001
Just before intubation	87.9 (14.4)	0.044**	73.7 (11.2)	<0.001**	<0.001
1 min post-intubation	90.2 (13.6)	0.136^#^	79.5 (9.5)	<0.001^#^	<0.001
2 min post-intubation	91 (14.4)	0.055^#^	78.1 (10.5)	<0.001^#^	<0.001
3 min post-intubation	90.6 (14)	0.107^#^	77.2 (11.2)	0.002^#^	<0.001
4 min post-intubation	89.2 (14)	0.467^#^	76.5 (12.1)	0.009^#^	<0.001
5 min post-intubation	87.5 (13.5)	0.795^#^	75.4 (12.4)	0.117^#^	<0.001
After LA infiltration	86.2 (13.8)		73.7 (11)		<0.001
Microscope on	79.3 (12.7)		67 (9.5)		<0.001
Microscope off	75.6 (10.5)		67 (10.3)		<0.001
Last skin suture	76.1 (10.9)		67 (10)		<0.001
Before mastoid dressing	77.5 (11.1)		67.8 (10.1)		<0.001
After mastoid dressing	78.5 (10.6)		69.3 (9.3)		<0.001
Extubation	81.2 (11.3)		68.3 (8.6)		<0.001
1 min post-extubation	82.9 (10.7)		70.9 (9.1)		<0.001
3 min post-extubation	83.1 (11)		71.5 (9.7)		<0.001

Baseline MAP was comparable in both groups (p=0.471). MAP for up to 5 min post-intubation was significantly higher in Group PD when compared to Group P (p<0.05). MAP was higher in Group PD through most of the intraoperative period, but this difference was not statistically significant. At extubation and up to 3 min post-extubation, MAP was significantly lower in Group PD (p<0.05) when compared to Group P. For within-group comparisons, MAP just before intubation, when compared to the respective baseline values, was significantly lower in both groups. When compared to MAP just before intubation, the post-intubation values were significantly higher in Group PD for up to 2 min (p<0.05). There was no significant change in post-intubation MAP in Group P when compared to MAP just before intubation (Table 4).

**Table 4:** Comparison of mean arterial pressure (mmHg) between groups LA: Local anaesthesia. Values presented as means (standard deviations). p-value comparisons are between the two groups (unpaired t-test). p-values* for within-group comparison (paired t-test), **when compared to baseline, and #when compared to just before intubation.

	Group P	p-value*	Group PD	p-value*	p-value
Baseline	97.4 (8.6)		95.8 (13.1)		0.471
After loading dose	93.7 (9.1)		93.9 (11.7)		0.954
Induction	87.3 (12.4)		88.3 (12.2)		0.674
Just before intubation	83.8 (15.4)	<0.001**	88 (14.4)	0.001**	0.167
1 min post-intubation	89.4 (12.4)	0.052^#^	96.4 (12.6)	<0.001^#^	0.006
2 min post-intubation	86.7 (12.7)	0.288^#^	92.8 (13.2)	0.005^#^	0.021
3 min post-intubation	84.5 (11.5)	0.773^#^	89.9 (12.9)	0.228^#^	0.029
4 min post-intubation	81.5 (8.8)	0.290^#^	88.5 (12)	0.727^#^	0.001
5 min post-intubation	81.4 (8.3)	0.297^#^	86 (12)	0.17^#^	0.028
After LA infiltration	79.6 (9.4)		83.7 (12.3)		0.060
Microscope on	71.5 (7.1)		75.8 (10)		0.015
Microscope off	81.2 (9.5)		83.7 (9.8)		0.209
Last skin suture	82.6 (9.5)		81.7 (9.8)		0.636
Before mastoid dressing	84.2 (9.7)		80.5 (8.9)		0.047
After mastoid dressing	85.9 (10.4)		81.7 (9.8)		0.038
Extubation	89 (10.8)		78.2 (8.9)		<0.001
1 min post-extubation	88.9 (9.1)		81.8 (8.7)		<0.001
3 min post-extubation	87.5 (8.1)		81.2 (10.2)		0.001

The time duration of MAP within the target range of 60-69 mmHg was higher in Group P when compared with Group PD, but this difference was not statistically significant (p=0.294) (Table 5).

**Table 5:** Duration of mean arterial pressure at 60-69 mmHg, 70-79 mmHg, and 81-89 mmHg MAP: Mean arterial pressure. Values are presented as means (standard deviations)

	Group P (n=50)	Group PD (n=50)	p-value
Duration of MAP 60–69 mmHg (min)	38.2 (36.8)	30.3 (38.1)	0.294
Duration of MAP 70–79 mmHg (min)	44.1 (40.2)	39.1 (33.1)	0.499
Duration of MAP 80–89 mmHg (min)	37.6 (43.2)	42.4 (31.2)	0.526

In addition, Group PD had a significantly better surgical field than that of Group P (p=0.000273).

The majority of patients in Group PD had a significantly deeper level of sedation when compared to Group P at the time of arrival (p=0.008) and at 30 min (p=0.0002) in the PACU. However, at 1 h and 2 h in the PACU, the level of sedation was comparable between the two groups (Table 6).

**Table 6:** Ramsay sedation scores PACU: post-anesthesia care unit. Values are presented as numbers (percentages). *Inter-group comparison. Ramsay sedation score: patient anxious or agitated or both=1; cooperative, oriented, and tranquil=2; responds to commands only=3; a brisk response to a light glabellar tap=4; sluggish response to a light glabellar tap=5; and no response=6

Ramsay sedation score	Group P (n=50)	Group PD (n=50)
On arrival to PACU* (p=0.008)		
1	0	0
2	0	0
3	39 (78)	25 (50)
4	0	0
5	11 (22)	22 (44)
6	0	3 (6)
30 min after arrival to PACU* (p=0.0002)		
1	0	0
2	47 (94)	28 (56)
3	3 (6)	20 (40)
4	0	1 (2)
5	0	1 (2)
6	0	0
1 h after arrival to PACU		
1	0	0
2	50 (100)	49 (98)
3	0	0
4	0	0
5	0	1 (2)
6	0	0
2 h after arrival to PACU		
1	0	0
2	50 (100)	49 (98)
3	0	1 (2)
4	0	0
5	0	0
6	0	0

Two patients in Group PD and none in Group P had an NRS of >3 for pain in the PACU. Both of these patients received intravenous morphine 0.05 mg/kg.

One patient in Group P developed an MAP of <60 mmHg following induction of anesthesia and was treated with intravenous ephedrine 6 mg. None of the patients required atropine or developed any complications such as PONV (hence no ramosetron) or respiratory depression during their PACU stay.

## Discussion

Our study was designed to evaluate the effects of addition of dexmedetomidine to propofol anesthesia in patients undergoing elective middle-ear surgery. We found that there was no statistically significant difference in the consumption of propofol between Group P and Group PD. The induction dose of propofol was significantly lower in Group PD. These findings are consistent with those of another study, which compared dexmedetomidine versus placebo with respect to the amount of propofol and morphine used for Bispectral Index (BISTM)-guided sedation and analgesia in mechanically ventilated, ICU patients after surgery [6]. The authors observed that the total amount of propofol required by the dexmedetomidine group was less than that needed by the placebo group. However, the difference was not statistically significant.

Studies have shown that patients who receive dexmedetomidine exhibit a significant decrease in intraoperative and postoperative HR and MAP [7,8]. In our study, at various points of assessment following the completion of the dexmedetomidine loading dose, there was a significant decrease in HR from baseline in Group PD when compared with Group P. However, at no point in time after the completion of the loading dose did the mean HR rise above the baseline mean HR in Group PD. The maximum rise of HR in Group PD was at 1 min post-intubation (Table 3).

When compared with the baseline, MAP just before intubation was significantly lower in both groups. Patients in Group P showed no significant change in MAP post-intubation when compared with those just before intubation, whereas patients in Group PD showed a significant rise in MAP up to 2 min post-intubation when compared with that just before intubation. There was no significant decrease in MAP following the completion of dexmedetomidine infusion or following induction of anesthesia in Group PD when compared with Group P. However, MAP in Group PD was significantly higher following intubation up to 5 min when compared to Group P. MAP up to 3 min post-extubation was significantly lower in Group PD when compared to Group P (Table 4). These changes in HR and MAP could be due to the reduced induction dose of propofol in Group PD.

In our study, Group PD had a significantly better surgical field, although there was no significant decrease in MAP at various points of assessment during microscope use. Nasreen and colleagues showed that the dexmedetomidine group in their study had a better surgical field when compared to the placebo group [4]. These findings are attributed to the fact that dexmedetomidine reduces sympathetic activity, resulting in a lower BP and HR, thereby decreasing blood loss at the surgical site and improving the quality of the operative field. Dexmedetomidine also has the property of peripheral vasoconstriction, similar to the local infiltration of adrenaline.

We observed a significantly prolonged recovery time in Group PD when compared with Group P. Other studies have also shown that dexmedetomidine prolongs recovery and causes delayed recovery room discharge [9, 10].

Yildiz et al. demonstrated the effect of single-dose preoperative dexmedetomidine on hemodynamic responses to laryngoscopy and intubation, perioperative hemodynamics, and anesthetic requirements, and concluded that dexmedetomidine reduced opioid and anesthetic requirements [11]. Bakhamees et al. showed that dexmedetomidine decreased pain scores and patient-controlled analgesia morphine use [1]. Dexmedetomidine, as a substitute for remifentanil in ambulatory gynecologic laparoscopy, demonstrated a decrease in perioperative opioid requirement [9]. In our study, two patients in Group PD and none in Group P showed an NRS of >3; both had an NRS of 7, one at 1 h and the other at 2 h after arrival to the PACU.

Our study showed that the majority of patients in Group PD had a significantly deeper level of sedation when compared to Group P at the time of arrival and at 30 min in the PACU. In a study conducted to evaluate the efficacy, side effects, and recovery characteristics of dexmedetomidine for intraoperative sedation, it was observed that dexmedetomidine produced deeper sedation scores than propofol during recovery [12]. The absence of PONV in our study could be due to the antiemetic properties of propofol. Dexmedetomidine has an opioid-sparing effect and leads to less morphine-induced nausea [13,14].

Our study did have some limitations. First, the assessment of the surgical field by the surgeon was subjective as there was no standard tool applied for the same. Also, there were ten Otorhinolaryngologists who operated on the patients; thereby, the surgical skills and expertise may also be a confounding factor that was difficult to eliminate in our methodology. Second, the depth of anesthesia was not monitored, as BISTM/EntropyTM were not available at that time in our institution. The anesthesiologist had the liberty to titrate the propofol infusion (50-150 μg/kg/min) to achieve the target MAP rather than depending on the depth of anesthesia. Therefore, monitoring the depth of anesthesia could have provided for better titration of propofol, and could have had an effect on propofol consumption. Finally, the blinding of the observer may not have been complete due to the fact that dexmedetomidine reduces HR and causes sedation, which the attending anesthesiologist may have noticed even though being blinded to the study.

To summarize, dexmedetomidine, due to its central sympatholytic effect, blunts haemodynamic responses in the perioperative period. It is said to potentiate the action of opioids and analgesics, thereby reducing the need for large doses of opioids and anaesthetics. The practice of middle ear surgery under GA requires hypotensive anaesthesia and a relatively bloodless field. Alpha-2 agonists like clonidine and dexmedetomidine have been used to achieve hypotensive anaesthesia. This study intended to evaluate the effect of the addition of dexmedetomidine on the requirement of propofol, intraoperative haemodynamic stability, quality of the surgical field, recovery time, postoperative opioid consumption, and adverse events like PONV and respiratory depression.

One hundred ASA PS 1 or 2 patients of either sex in the age group 18-60 years undergoing middle ear surgery under GA were enrolled in this prospective randomized double-blind observational study.

Demographic data were comparable between the two groups. There was no significant difference in the consumption of propofol between the two groups, whereas the induction dose of propofol was significantly lower in group PD. Group PD showed a statistically significant decrease in the HR during the perioperative period, whereas MAP did not remain within the target range of 60-70 mmHg for a longer time in group PD when compared with group P (statistically not significant). Patients in group PD had an excellent surgical field. When compared with group P, patients in group PD had significantly prolonged recovery and had higher sedation scores as assessed by the Ramsay Sedation Assessment Scale. None of the patients in the study developed any adverse events like PONV, respiratory depression, etc. in the PACU.

## Conclusions

Intravenous dexmedetomidine infusion does not reduce intraoperative propofol consumption but reduces the induction dose in patients undergoing middle-ear surgery. However, dexmedetomidine decreases HR significantly without causing a significant reduction in MAP during the intraoperative period, and provides a significantly better surgical field compared to propofol alone. Infusion of dexmedetomidine in doses used in our study delays recovery significantly.

## References

[1] Bakhamees HS, El-Halafawy YM, El-Kerdawy HM, Gouda NM, Altemyatt S (2007). Effects of dexmedetomidine in morbidly obese patients undergoing laparoscopic gastric bypass. Middle East J Anaesthesiol.

[2] Sudheesh K, Harsoor S (2011). Dexmedetomidine in anaesthesia practice: a wonder drug?. Indian J Anaesth.

[3] Degoute CS (2007). Controlled hypotension: a guide to drug choice. Drugs.

[4] Nasreen F, Bano S, Khan RM, Hasan SA (2009). Dexmedetomidine used to provide hypotensive anesthesia during middle ear surgery. Indian J Otolaryngol Head Neck Surg.

[5] Yazbek-Karam VG, Aouad MM (2006). Perioperative uses of dexmedetomidine. Middle East J Anaesthesiol.

[6] Triltsch AE, Welte M, von Homeyer P (2002). Bispectral index-guided sedation with dexmedetomidine in intensive care: a prospective, randomized, double blind, placebo-controlled phase II study. Crit Care Med.

[7] Wahlander S, Frumento RJ, Wagener G, Saldana-Ferretti B, Joshi RR, Playford HR, Sladen RN (2005). A prospective, double-blind, randomized, placebo-controlled study of dexmedetomidine as an adjunct to epidural analgesia after thoracic surgery. J Cardiothorac Vasc Anesth.

[8] Venn RM, Grounds RM (2001). Comparison between dexmedetomidine and propofol for sedation in the intensive care unit: patient and clinician perceptions. Br J Anaesth.

[9] Salman N, Uzun S, Coskun F, Salman MA, Salman AE, Aypar U (2009). Dexmedetomidine as a substitute for remifentanil in ambulatory gynecologic laparoscopic surgery. Saudi Med J.

[10] Alhashemi JA (2006). Dexmedetomidine vs midazolam for monitored anaesthesia care during cataract surgery. Br J Anaesth.

[11] Yildiz M, Tavlan A, Tuncer S, Reisli R, Yosunkaya A, Otelcioglu S (2006). Effect of dexmedetomidine on haemodynamic responses to laryngoscopy and intubation : perioperative haemodynamics and anaesthetic requirements. Drugs R D.

[12] Arain SR, Ebert TJ (2002). The efficacy, side effects, and recovery characteristics of dexmedetomidine versus propofol when used for intraoperative sedation. Anesth Analg.

[13] Massad IM, Mohsen WA, Basha AS, Al-Zaben KR, Al-Mustafa MM, Alghanem SM (2009). A balanced anesthesia with dexmedetomidine decreases postoperative nausea and vomiting after laparoscopic surgery. Saudi Med J.

[14] Lin TF, Yeh YC, Lin FS, Wang YP, Lin CJ, Sun WZ, Fan SZ (2009). Effect of combining dexmedetomidine and morphine for intravenous patient-controlled analgesia. Br J Anaesth.

